# Enhancing Therapeutic Drug Monitoring in Inflammatory Bowel Disease: A Comparative Analysis of Rapid Point-of-Care Infliximab, Adalimumab and Anti-Drug Antibodies’ Determination against ELISA

**DOI:** 10.3390/pharmaceutics15112615

**Published:** 2023-11-11

**Authors:** Francisco José Toja-Camba, Laura García-Quintanilla, Lorena Rodríguez-Martinez, Julia Tomine, Francisco Cajade-Pascual, Carolina Feitosa, Irene Zarra-Ferro, Manuel Barreiro-De-Acosta, Jaime González-López, Cristina Mondelo-García, Anxo Fernández-Ferreiro

**Affiliations:** 1 Pharmacy Department, University Clinical Hospital of Santiago de Compostela (SERGAS), 15706 Santiago de Compostela, Spain; kikotoja@gmail.com (F.J.T.-C.); lauragarqu@gmail.com (L.G.-Q.); francajade13@gmail.com (F.C.-P.); irene.zarra.ferro@sergas.es (I.Z.-F.); jaime.gonzalez.lopez@sergas.es (J.G.-L.); 2 Clinical Pharmacology Group, Health Research Institute of Santiago de Compostela (IDIS), 15706 Santiago de Compostela, Spain; lorenamarinoalvarez@gmail.com (L.R.-M.); carolinafeimed@gmail.com (C.F.); 3 Faculty of Pharmacy, University of Santiago de Compostela, 15782 Santiago de Compostela, Spain; 4 Pharmacy Department, Faculty of Health, University of Angers, 16, Boulevard Daviers, 49045 Angers, France; jtomine0@gmail.com; 5 Gastroenterology Department, University Clinical Hospital of Santiago de Compostela (SERGAS), 15706 Santiago de Compostela, Spain; manuel.barreiro.de.acosta@sergas.es

**Keywords:** point-of-care, infliximab, adalimumab, anti-drug antibodies, ELISA

## Abstract

The introduction of point-of-care (POC) assays into clinical practice in patients with inflammatory disease enables on-demand therapeutic decision making. The aim of this study was to compare the POC test Quantum blue (Bühlmann Laboratories) for infliximab (IFX), adalimumab (ADL), and its anti-drug antibodies with the traditional ELISA assay (Promonitor). A total of 200 serum samples were analyzed. Samples were classified into the following three different groups; sub-therapeutic range (IFX < 3 μg/mL and ADL < 5 μg/mL); therapeutic range (IFX: 3–7 μg/mL and ADL: 5–12 μg/mL) and supra-therapeutic range (IFX levels > 7 μg/mL and ADL levels > 12 μg/mL). Significant higher values were measured using the POC test (*p* < 0.001) for IFX results but no differences in ADL trough levels were observed (*p* = 0.3101). Spearman’s correlation indicated a good correlation between the two assays (rs = 0.88 for ADL and rs = 0.93 for IFX), and McNemar’s test revealed significant differences (*p* = 0.016) when classifying IFX samples between therapeutic and supra-therapeutic ranges but no significant differences were found among the other ranges for either IFX or ADL. These results show that we should be cautious when using these rapid measurement methods, and new targets should probably be defined for IFX when using this new analytical method.

## 1. Introduction

Inflammatory bowel disease (IBD) is a chronic immune-mediated inflammatory disease affecting the gastrointestinal tract that englobes two entities, Crohn’s disease (CD) and ulcerative colitis (UC), which are both characterized by recurrent and destructive pathological inflammation that causes significant morbidity and impacts the quality of life. While it is currently an incurable disease, there are several treatments that target clinical symptoms. The introduction of biologic drugs has revolutionized the approach to the treatment of IBD. This has led to improved and timely treatment responses, lower hospitalization rates, reduced surgical requirements, and remarkable outcomes, such as complete mucosal histologic healing and an improvement in quality of life [1]. Anti-tumor necrosis factor (TNF) therapies are the cornerstone of the treatment for IBD and other chronic inflammatory conditions, including rheumatoid arthritis and psoriasis [2]. Infliximab (IFX) and adalimumab (ADL) are monoclonal antibodies (chimeric for IFX and fully human for ADL) that target the TNF alpha proinflammatory cytokine [3,4]. IFX was the first monoclonal antibody approved for the treatment of IBD in both CD and UC. IFX is a chimeric (human 75% and murine 25%) monoclonal IgG1-type antibody that is directed against the soluble and cell membrane TNF-α, which fixes the complement, promotes antibody-mediated cytotoxicity and induces T-cell apoptosis. Several years later, IFX was followed by ADL, which is a recombinant monoclonal antibody (100% human) directed against TNF-α. It neutralizes its biological function by blocking its interaction with p55 and p75 receptors on the cell surface and attenuating its proinflammatory effects. Their introduction as the first line of biological therapy was a great advantage for IBD patients because they were shown to induce and maintain both clinical remission and mucosal healing when conventional therapies became unresponsive, and they significantly improved patients’ quality of life [5,6].

The discovery of new mechanisms of action and the optimization of conventional treatments has greatly improved the situation; however, approximately 20–30% of patients with IBD show a primary non-response to biologic therapies, and up to 50% of patients discontinue treatment because of a secondary loss of response or a serious adverse event after an initial clinical response [1]. Many studies show a correlation between how low or undetectable drug concentrations lead to immunogenicity and treatment failure [7]. Also, the formation of anti-drug antibodies (ADAs) is associated with a loss of response by accelerating drug clearance through complex formations and also with the increased risk of infusion reaction [8,9]. The incidence of anti-infliximab antibodies ranges widely from 6.1 to 73% [5] and nearly 35% for ADL [10]. A prospective observational study found that 75% of patients developed ADAs to IFX by week 22, and 90% of patients developed ADAs to IFX within the first 12 months of therapy, some of which are transient antibodies that have no clinical significance [11].

Therapeutic drug monitoring (TDM), defined as the assessment of drug concentrations and the detection of ADAs, is an important tool for optimizing biologic therapy [6]. In a reactive strategy, TDM can be used to identify the cause for the secondary loss of response. In patients with low trough levels observed during plasma monitoring and those with undetectable levels or low ADAs, the most successful therapeutic strategy is to increase the dose of anti-TNF drugs because they have insufficient drug concentrations. By contrast, patients with a secondary loss of response and high circulating levels of anti-TNF antibodies do not respond to increasing doses of anti-TNF because ADAs increase clearance by binding to the circulating drug, causing the neutralization of the drug’s effects [12]. For patients with pharmacodynamic failure, trough levels of anti-TNF are within the optimal therapeutic range, and this may indicate that the ongoing inflammatory process is independent of anti-TNF signaling. Reactive TDM has not only been shown to lead to the earlier targeting of the most effective treatment and avoidance of potentially unnecessary drug exposure, but it is also a more cost-effective method [13,14]. As opposed to reactive TDM, the utility of proactive TDM, usually performed to decrease the likelihood of and secondary loss of response while the patient is in remission, is controversial throughout the available literature. Since the publication of the TAXIT study [15], many studies have analyzed the benefits of TDM, although the results are not always consistent. Since 2017, multiple guidelines for TDM in IBD have been published. Specifically, both the American Gastroenterological Association [16] and the Gastroenterological Society of Australia [10] recommend the use of reactive TDM for ongoing active inflammation based on different tests to guide treatment changes, but they do not recommend proactive TDM before treatment failure.

The analysis of blood concentrations of monoclonal antibodies is performed routinely using the enzyme-linked immunosorbent assay (ELISA). This requires the logistic accumulation of samples that are necessary to make it a cost-efficient technique, which is why these methods are commonly centralized. However, it does not lend itself to prompt decision making, often delaying clinical decisions that demand precision, agility, and practicality. Point-of-care (POC) tests allow the measurement of IFX and ADL through concentrations and the rapid detection of ADAs on the same day of consultation, providing instant results in less than half an hour. POCs are rapid immunochromatographic assays based on lateral flow technology for the quantitative detection of drugs and qualitative detection in the case of ADAs. Currently, there are three rapid detection tests that are commercialized: the Promonitor Quick assay (PQ) (Grifols Diagnostic, Emeryville, CA, USA), Quantum Blue^®^ (QB) (Bühlmann Laboratories, Schönenbuch, Switzerland), and Rida^®^Quick (RQ) (RBiopharm, Darmstadt, Germany). They vary in their range of detection in the samples required for detection (serum/plasma/whole blood).

Comparative studies between both methods are needed to provide solid evidence to incorporate POCs into daily clinical practice and, thus, reduce response times. In addition, it is important to elucidate whether the therapeutic ranges used, based primarily on ELISA results, are also applicable to these new rapid methods. There are some published studies on a short series of samples [4,17,18,19,20] in which the different POCs have been compared with ELISA as the current gold standard.

The aim of this study is to compare the POC test Quantum Blue for quantitative IFX and ADL and qualitative anti-IFX and anti-ADL analysis with the traditional ELISA assay (Promonitor) to evaluate their concordance in the measurement of trough levels, the detection of antibodies, and whether using one method or the other implies changes in therapeutic decisions.

## 2. Materials and Methods

This was a retrospective study in which patients with IBD were selected, and their ADL and IFX trough levels were monitored following the hospital protocol using the ELISA technique. The study and the data collection strictly adhered to the declaration of Helsinki principles. Ethical approval was given by the local Institutional Review Board and the autonomous region of Galicia (2018/077).

### 2.1. Samples and Recollection

**Figure 1:**
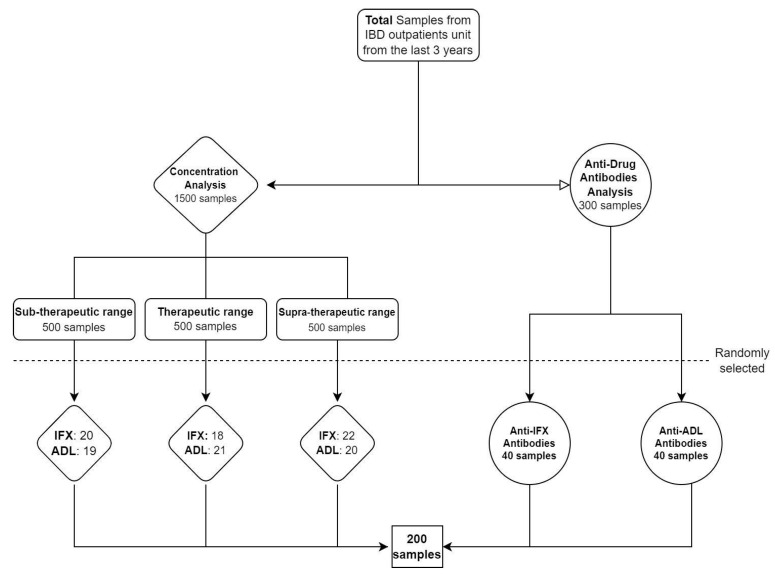
Sampling methodology.

A total of 200 serum samples (60 ADL, 60 IFX, 40 anti-IFX, and 40 anti-ADL) from 200 different patients were analyzed from the IBD unit. One thousand five hundred samples for concentration analysis were classified into three different groups according to their sub-therapeutic (IFX levels < 3 μg/mL and ADL levels < 5 μg/mL), therapeutic (IFX levels 3–7 μg/mL and ADL levels 5–12 μg/mL) and supra-therapeutic (IFX levels > 7 μg/mL and ADL levels > 12 μg/mL) ranges, and samples from the total amount of each group were randomly selected. Also, 40 samples were randomly selected for anti-IFX and 40 samples for anti-ADL analysis (Figure 1).

#### 2.1.1. Quantitative ELISA Analysis

Promonitor is a capture (IFX kit) or sandwich (ADL kit) ELISA in which microwell strips are provided and pre-coated with an anti-TNF human F(ab’)2 fragment bound to a recombinant human TNF. Both Promonitor anti-IFX and anti-ADL bridge ELISA in which microwell strips are provided and pre-coated with IFX and ADL, respectively. Samples were centrifuged at 2280× *g* for 10 min within 4 h after sample collection and stored at −80 °C before analysis. ELISA analyses were performed following routine clinical practice with the Promonitor ELISA kit according to the manufacturer’s instructions (Promonitor, Grifols Diagnostic, Barcelona, Spain), and all were run on a semi-automated ELISA processor (Triturus^®^, Grifols). The dilution of the serum samples was modified to 1:400 and 1:10 for IFX and ADL trough levels and to 1:1 and 1:10 for IFX and ADL ADA. For quantitative analysis, according to the package insert (Promonitor, Grifols Diagnostic, Barcelona, Spain) the lower limit of quantification (LLoQ) for ADL was 0.017 μg/mL, and the LLoQ for Promonitor-IFX was determined to be 0.3 μg/mL. In the case of the ADA assay, the LLoQ of Promonitor anti-IFX was determined to be 2 AU/mL, and the LLoQ of Promonitor anti-ADL was determined to be 3.7 AU/mL.

#### 2.1.2. Quantitative POC Analysis

Quantum Blue^®^ (QB) is an in vitro diagnostic lateral flow immunoassay for the quantitative determination of trough levels of ADL and IFX and qualitative determination of ADAs detection in serum samples (Bühlmann Laboratories Schönenbuch, Schönenbuch, Switzerland), POC analysis was made following the manufacturer’s instructions. Briefly, serum samples for the determination of IFX and ADL were diluted with an assay buffer (1:20). For high-concentration samples, an additional dilution of 1:200 was made. In the case of ADA analysis, the dilution was 1:10. Then, diluted serum samples were incubated for 15 min at room temperature before the results were interpreted by the Quantum Blue Reader (Bühlmann Laboratories Schönenbuch, Switzerland). According to the package insert, the lower limit of quantitation for QB-ADL was 1.3 μg/mL, and the LLoQ for QB-IFX was 0.32 μg/mL. As QB only shows qualitative results for ADA analysis, the cut-off point was set at 0.2 μg_eq_/mL for the QB anti-ADL assay and 1.3 μg_eq_/mL for the QB anti-IFX assay to distinguish between negative and positive results.

### 2.2. Statistical Analysis

Normality was tested using the Shapiro–Wilk test and correlation was evaluated with Spearman’s rank correlation coefficient (rs). The Wilcoxon rank sum test was used to detect differences between the drug trough levels. A passing-Bablock regression was performed to estimate the line of best fit by comparing the ranks of observations between the two variables. Also, Bland–Altman analysis was conducted to assess the agreement between the two quantitative measurements. A Bland–Altman plot showed the difference between these two measurements (y-axis), and its mean (x-axis) represented the mean difference between the two measurements, and the limits of agreement were expressed as the mean difference plus or minus the two standard deviations of differences. The concordance between the ELISA and QB classification of patients was placed into three different therapeutic groups for both drugs assessed through weighted Cohen’s kappa (κ), and the differences in classification for each group were assessed using McNemar’s test. Also, the concordance between the detection of ADAs with ELISA and QB for both drugs was run using Cohen’s kappa (κ).

## 3. Results

### 3.1. Trough Levels

According to the classification of the different ranges, the 120 samples for quantitative analysis were distributed as follows: 20 samples for the IFX sub-therapeutic range, 19 for the ADL sub-therapeutic range, 18 samples for the IFX therapeutic range, 21 for the ADL therapeutic range, 22 samples for the IFX supra-therapeutic range and 20 for the ADL supra-therapeutic range.

ADL trough concentrations were measured using the two-method analysis in 60 samples. The median values were 10 µg/mL (IQR: 3.87–13.25) for the Promonitor assay and 8.85 µg/mL (IQR: 3.67–13.62) for the Quantum Blue assay. No differences in ADL trough levels were observed between the Promonitor and QB (*p* = 0.3101). Also, 60 samples of patients treated with IFX were measured via the Promonitor and Quantum Blue assays. The median values were 4.86 µg/mL (IQR: 2.22–9.31) for the Promonitor assay and 6.15 µg/mL (IQR: 2.9–12.92) for the Quantum Blue assay, with significantly higher values measured using the QB test (*p* < 0.001). Spearman’s rank correlation coefficient indicated a good correlation for ADL trough levels between the two assays (rs = 0.88), which was even higher between IFX trough levels (rs = 0.93).

**Figure 2:**
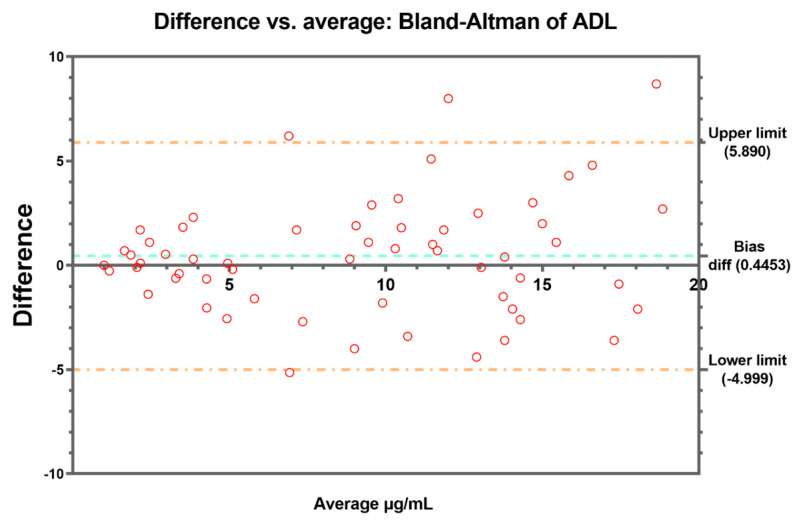
Bland–Altman’s plot. The difference in ADL concentrations vs. the average (µg/mL) between Promonitor and QB. The dashed blue line represents the bias and dashed orange lines represent the 95% limit of agreement.

**Figure 3:**
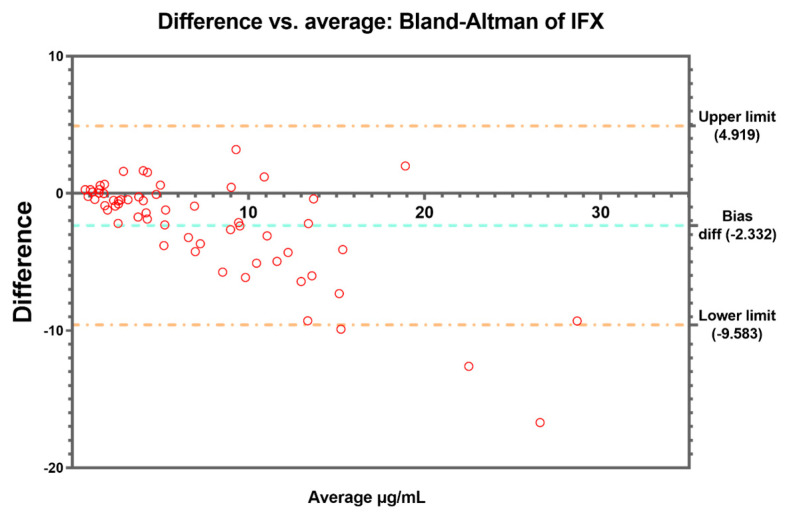
Bland–Altman’s plot. The difference in IFX concentrations vs. the average (µg/mL) between Promonitor and QB. The dashed blue line represents the bias and dashed orange lines represent the 95% limit of agreement.

**Figure 4:**
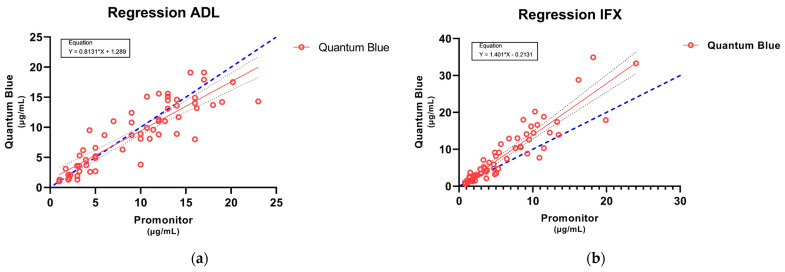
Passing-Bablock. The regression of measured concentrations between the Quantum blue and Promonitor kit for ADL (**a**) and IFX (**b**). The blue line represents the identity line. The grey dashed lines are the 95% confidence bounds.

Bland–Altman’s analysis was conducted to complete the comparison between the methods, revealing a bias difference of 0.4453 between them for ADL (Figure 2) and a bias difference of −2.332 in the case of IFX (Figure 3). Four and three values above the 95% limit of agreement were found for ADL and IFX, respectively. These results were corroborated by the regression performed to estimate the line of best fit comparing both methods (Figure 4a,b).

### 3.2. Stratification in Ranges

Stratifying results in sub-, supra- and therapeutic ranges was necessary due to their clinical importance and significance. Weighted Cohen’s kappa statistics revealed a substantial agreement for both molecules [21]; κ = 0.751 ± 0.063 for ADL (Table 1) and κ = 0.763 ± 0.059 for IFX (Table 2). Overall, the qualitative agreement between the two methods increased up to 78.33% for both drugs. In the case of ADL, for samples < 5 µg/mL, the agreement was 16/19 (84.21%); for the range 5–12 µg/mL, the agreement was 15/21 (71.43%), and for samples with concentrations above 12 µg/mL, the agreement was 16/20 (80%). On the other hand, for IFX trough levels under 3 µg/mL, the agreement was 15/20 (75%); for the therapeutic range (3–7 µg/mL), the agreement was 10/18 (55.56%), and for concentrations above 7 µg/mL, the agreement was 22/22 (100%).

The therapeutic strategy decision could be different in 13 samples (21.6%) for each treatment. To complete the analysis, McNemar’s test was performed to assess whether each method resulted in discordant classifications considering the sub-therapeutic and therapeutic range and therapeutic and supra-therapeutic range. McNemar’s test revealed that there were significant differences (*p* = 0.016) when classifying IFX samples between therapeutic and supra-therapeutic ranges, but no significant differences were found among the other ranges for either IFX or ADL (Table 1 and Table 2).

### 3.3. Anti-Infliximab and Anti-Adalimumab Antibodies

A total of 40 samples from IBD patients who received ADL and IFX and had sub-therapeutic levels were tested using the Promonitor kit and Quantum Blue assays. As Quantum Blue is a qualitative test for ADAs’ determination, a qualitative comparison was conducted between these two methods. A moderate agreement (κ = 0.536 ± 0.136) between the two assays was revealed for infliximab ADAs. In the case of adalimumab ADAs, the agreement was substantial (κ = 0.793 ± 0.095). Also, McNemar’s test was performed to complete the comparative classification, and no statistically significant differences were found for either anti-ADL (*p* = 0.1336) or anti-IFX (*p* = 0.0771) tests. Descriptive analysis and kappa statistics results are shown in Table 3.

## 4. Discussion

The management of inflammatory bowel disease (IBD) has advanced significantly in recent years, with the increasing availability of biologic agents, but compared to other immune-mediated pathologies, they are still insufficient [22]. Therefore, TDM is one of the most important keys to optimizing individual therapy. The measurement of serum anti-TNF concentrations is considered a useful tool to optimize treatment response and make quick and important clinical decisions in patients receiving anti-TNF therapy, preferably in combination with ADA testing. An important prerequisite to achieving this is to have an analytical method that allows not only a short turnaround time but also one that can simultaneously determine the concentration of anti-TNF and the presence of ADAs. Considering that the TDM strategy with the highest consensus on its usefulness and cost-effectiveness is reactive TDM, short response times become even more important. This monitoring can be performed at any patient visit and when a relapse is clinically observed, thus shortening therapy optimization times and future complications. In this sense, POCs have become more common in laboratories due to their accessibility, ease of use, and speed in obtaining results. Despite their importance, methodological comparisons between POC and ELISA techniques are needed to assess whether they can be interchangeable. Current evidence suggests that, despite good mathematical correlations between different tests, including the commonly used ELISA and POC tests, these latter tests may lead to different treatment decisions. To our knowledge, this is the first study to compare these two methods (QB and Promonitor) and the largest between POC methods and traditional ELISA assays, including a comparison of both IFX and ADL concentrations as well as ADAs.

In the case of ADL, our results show a good correlation and no significant differences in the measurement of trough levels between the two methods, as well as in the stratification of patients in different therapeutic ranges. This has been also observed in another study [23], where they found that the QB monitoring of ADL levels is a reliable and interchangeable alternative to the commonly used ELISA-based ADL quantification kits, although Promonitor was not among the kits compared. By contrast, the comparison of IFX concentrations between the two methods was unsatisfactory, with poor agreement in higher concentrations of IFX and large differences (−2.32) observed in Bland–Altman’s plot. On the one hand, this differs from the results of previous studies, but in one of these reports, as in those mentioned above, ELISA kits other than Promonitor were also used [17,19]. On the other hand, these results are in line with the trends observed by Dutzer et al. [24], as the largest differences were observed at the highest IFX concentrations, although, in this case, the ELISA kit used for comparison was different. However, this fact did not lead to relevant differences when classifying patients into different ranges and applying Cohen’s kappa test (K = 0.763). There is considerable disagreement as to whether the kappa statistic is useful for assessing the agreement between methods; nevertheless, it is used as one of the statistical methods of comparison. In this sense, the kappa statistic should not be considered as a definitive rule or a standard way to quantify concordance, and caution should be taken when using a statistic that has generated so much controversy. Despite the results of the weighted kappa indicating a moderate to substantial agreement in the classification of patients into different therapeutic ranges, when McNemar’s test was performed, it was observed that there were significant differences, specifically when classifying the samples into the therapeutic range and supra-therapeutic range for IFX. These results reaffirm the differences found in the highest IFX concentrations and the previously published results by Dutzer et al. [24].

In our study, QB systematically provided higher IFX trough values than those determined by ELISA, which led us to conclude that the target value of IFX values in TDM, according to different measurements, are different depending on the method. In our study, 5/20 (25%) patients with sub-therapeutic ranges measured by ELISA were classified as therapeutic by QB, and 7/18 (38.8%) in a therapeutic range by ELISA were classified as supra-therapeutic by QB. This led us to the fact that, in cases of doubt, it is always necessary to confirm with the reference method before taking any clinical decision and to be cautious to not rely solely on rapid measurement methods. Also, it may be necessary to redefine the target ranges of POC analytical methods, at least with the IFX test.

Finally, antibody detection using Promonitor ELISA kits is a robust method with high sensitivity and specificity, but, as with its drug testing counterpart, multiple samples need to be accumulated to make it cost-effective. The introduction of POCs into daily clinical practice would allow this to be resolved at the patient’s bedside in a few minutes; this is especially important in patients under ADL treatment because, due to their schedule of 40 mg every other week, physicians can make decisions before the next drug injection. In the case of antibody detection by both methods, no statistically significant differences were found for either anti-IFX or anti-ADL. One of the limitations of this assay is that we have not analyzed the effect of the presence of drugs in the sample, despite the fact that all of them came from sub-therapeutic levels or from other interfering substances in the detection of ADAs. In this sense, one prior study has already shown a correlation between these two analytical methods, but only regarding the detection of anti-IFX antibodies [25]. Lastly, it is necessary to highlight that our results are reinforced by the high number of samples analyzed and the fact that they all came from the same IBD unit and, therefore, the treatment of patients is homogeneous.

## 5. Conclusions

The routine TDM of ADL can be performed with Promonitor and QB assays since trough levels and patient classification into therapeutic ranges are comparable. In the case of IFX trough levels, we must be cautious when using these new methods due to the differences found when classifying patients between the therapeutic and supra-therapeutic ranges. New ranges should probably be defined for using different methods and not be considered interchangeable, especially when monitoring patients’ maintenance therapies. On the other hand, QB appears to be a suitable method for the qualitative determination of ADAs and could be used individually or as an add-on to the measurement of levels via traditional methods. Nevertheless, further studies with larger sample sizes and comparing all currently available analytical methods in a simultaneous manner are needed to establish the interchangeability between them and, if necessary, new therapeutic ranges according to the analytical method used.

## Figures and Tables

**Table 1:** Stratification of IBD patients between therapeutic ranges according to Promonitor and QB concentrations for ADL.

		Number of Samples (%)				Statistics		
		Quantum Blue				Cohen’s Weighted Kappa		
ADL Trough Concentrations		<5 µg/mL	5–12 µg/mL	>12 µg/mL	Total	K	SE	CI 95%
Promonitor	<5 µg/mL	16 (26.6)	3 (5)	0	19 (31.6)	0.751	0.063	0.626–0.876
	5–12 µg/mL	3 (5)	15 (25)	3 (5)	21 (35)	McNemar test		
	>12 µg/mL	0	4 (6.6)	16 (26.6)	20 (33.3)	(<5 µg/mL)-(5–12 µg/mL)	(5–12 µg/mL)-(>12 µg/mL)	
	Total	19 (31.6)	22 (36.6)	19 (31.6)	60 (100)	*p*-value = 1	*p*-value = 1	

**Table 2:** Stratification of IBD patients between therapeutic ranges according to Promonitor and QB concentrations for IFX.

		Number of Samples (%)				Statistics		
		Quantum Blue				Cohen’s Weighted Kappa		
IFX Trough Concentrations		<3 µg/mL	3–7 µg/mL	>7 µg/mL	Total	K	SE	CI 95%
Promonitor	<3 µg/mL	15 (25)	5 (8.3)	0	20 (33.3)	0.763	0.059	0.647–0.880
	3–7 µg/mL	1 (1.6)	10 (16.6)	7 (11.6)	18 (30)	McNemar test		
	>7 µg/mL	0	0	22 (36.6)	22 (36.6)	(<3 µg/mL)-(3–7 µg/mL)	(3–7 µg/mL)-(>7 µg/mL)	
	Total	16 (26.6)	15 (25)	29 (48.3)	60 (100)	*p*-value = 0.219	*p*-value = 0.016	

**Table 3:** Distribution of positive and negative patients to anti-IFX and anti-ADL antibodies measured with Promonitor and QB assays.

	Number of Samples (%)						
	Quantum Blue				Kappa Statistics		
Anti-IFX Antibodies		Positive	Negative	Total	K	SE	CI 95%
Promonitor	Positive	8 (20)	7 (17.5)	15 (37.5)	0.536	0.136	0.269–0.803
	Negative	1 (2.5)	24 (60)	25 (62.5)			
	Total	9 (22.5)	31 (77.5)	40 (100)			
	**Number of Samples (%)**						
	**Quantum Blue**				**Kappa Statistics**		
**Anti-ADL Antibodies**		**Positive**	**Negative**	**Total**	**K**	**SE**	**CI 95%**
Promonitor	Positive	14 (35)	0	14 (35)	0.793	0.095	0.606–0.981
	Negative	4 (10)	22 (55)	26 (65)			
	Total	18 (45)	22 (55)	40 (100)			

## Data Availability

Data are contained within the article.
